# Valsalva purpura on the face of a 7-year-old child

**DOI:** 10.25122/jml-2023-0447

**Published:** 2023-12

**Authors:** Alia Ali Galadari, Moteb Al Otaibi

**Affiliations:** 1 Department of Medicine, Dubai Health, Dubai, UAE; 2 Department of Medicine, Dermatology, Unaizah College of Medicine, Qassim University, KSA

**Keywords:** Valsalva maneuver, petechiae, purpura, Valsalva purpura, bruising

## Abstract

Valsalva purpura refers to purpura resulting from performing the Valsalva maneuver, a forced expiratory effort against a closed glottis. There are limited reported cases of Valsalva purpura in children, specifically on the cheeks. We present the case of a 7-year-old child who developed Valsalva purpura on his cheeks after performing the Valsalva maneuver during deep breathing exercises by holding his breath underwater during his swimming sessions. This article overviews the relationship between the Valsalva maneuver, purpura, and similar cases.

## INTRODUCTION

Purpura is characterized by a visible hemorrhage into the skin and mucous membranes. It does not usually blanch with pressure. It can indicate various hereditary or acquired diseases, such as clotting factors, platelet defects, and connective tissue disorders [1]. Valsalva maneuver is forced expiration against a closed glottis. This includes some activities we usually perform, such as balloon blowing and straining during defecation [2]. Valsalva maneuver causes an increase in the internal body pressure, which may lead to dermal capillary rupture. This can manifest as purpura or petechiae on the skin [3]. The cases of Valsalva purpura occurring on the face are rarely reported, specifically in pediatric literature. One case report presented a case of a 12-year-old girl who developed perioral petechiae due to dermal capillary rupture as a result of blowing up multiple balloons [3]. Another case report also discussed the appearance of bilateral infraorbital purpura in a 10-year-old girl after blowing a balloon [4]. In our patient, the 7-year-old child was trying to hold his breath underwater, which showed a correlation between the Valsalva maneuver and a characteristic purpura on his cheeks.

## CASE REPORT

**Figure 1:**
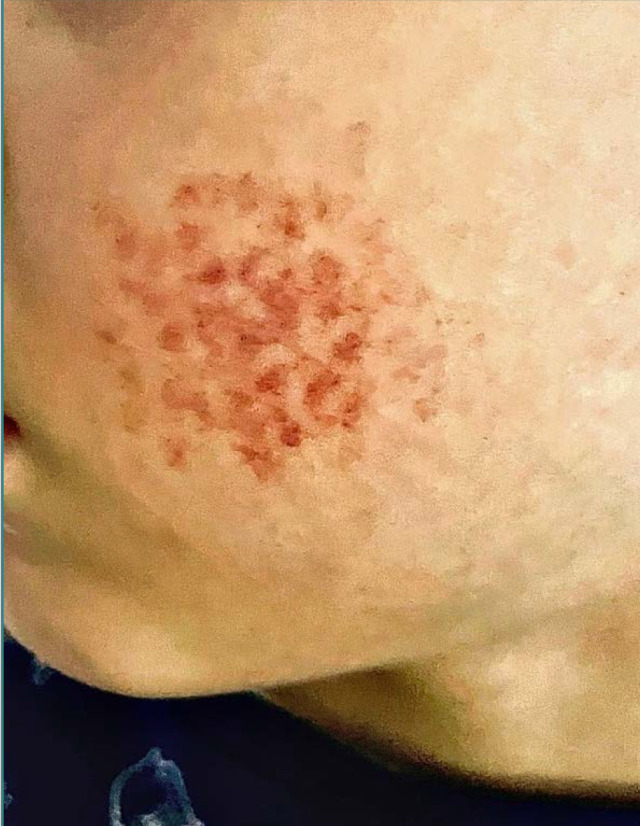
Multiple purpuric macules on the left cheek

A 7-year-old boy was brought by his parent to the dermatology clinic to evaluate a skin rash that had appeared on both his cheeks three days earlier. The boy was described as a healthy, playful child who enjoys playing and living a normal life. Three days earlier, the child’s family noticed the development of sudden red rashes on both cheeks (Figure 1). There were no local or systemic symptoms associated with the rash. This was the first time the boy developed such a skin condition, and he had no known medical conditions or blood disorders. The detailed history taken from the child and his father revealed that the boy joined a swimming class two weeks before this incident. According to his father, the child was trying to learn to take a deep breath and hold it during his dive. He did this repetitively to score higher seconds and minutes with each dive. There was no history of any systemic diseases, trauma, vomiting, straining, or medication use. Family history was unremarkable.

## PHYSICAL EXAMINATION, SKIN EXAMINATION, AND LAB RESULTS

Examination revealed a normal, healthy child with normal vital signs. He had bilateral, symmetrical, grouped red macules with 4-10 mm diameter (Figure 1). The lesions were non-palpable, non-blanching, and non-tender. The boy had no other lesions in the hair, nails, or mucosal areas. Full body skin exam was nonremarkable.

The investigations done to exclude any potential bleeding disorders included complete blood count (CBC), partial thromboplastin time (PTT), prothrombin time (PT), bleeding time, and comprehensive metabolic panel (CMP). All results were within the normal limits.

After reviewing the case and the lab results, the boy was diagnosed with purpura resulting from performing the Valsalva maneuver by holding his breath underwater. He was treated conservatively, meaning the father was advised to apply an ice pack wrapped in a soft cloth on the affected area for 15 to 20 minutes every few hours. The father was also advised to monitor his son’s condition and visit back the clinic in case the purpura resumed or got worse or if it appeared in other areas. However, the boy did fine, and his rash disappeared within one week.

## DISCUSSION

### Purpura

Purpura is a term that refers to discoloration of the skin due to the leakage of underneath blood vessels [5]. It usually occurs secondary to the excavation of red blood cells into the dermis or mucous membranes [3]. The causes of purpura can be further classified into platelet disorders (e.g., thrombocytopenic purpura), other coagulation disorders (e.g., disseminated intravascular coagulation), and vascular disorders (e.g., damage to blood vessels, increased intraluminal pressure). Identifying the cause of purpura on the skin is essential to initiate the appropriate treatment and management plan [5].

### The development of purpura due to the Valsalva maneuver

As explained above, the Valsalva maneuver is the forced expiration against a closed glottis. It is generally considered safe to do. However, it raises the intrabdominal pressure and produces marked changes in blood pressure. Therefore, it can have some risks and complications. These include Valsalva retinopathy, syncope, chest pain, arrhythmias, and spontaneous purpura [2]. Valsalva purpura is a term that describes purpura secondary to activities that imply forced pressure. Multiple activities, some of which include coughing, sneezing, vomiting, and powerlifting, have been reported with Valsalva purpura [6-9]. This usually occurs on the periocular area, neck, and upper chest. Valsalva purpura on the face has been rarely reported in the pediatric literature [2]. Valsalva purpura results from ruptured capillaries in the subcutaneous tissues and blood extravasation in response to increased capillary pressure [10]. We presented the case of a patient who developed purpura on the cheek area after performing the Valsalva maneuver while holding his breath underwater. During this maneuver, the patient drastically increased intracapillary pressure, causing the capillaries to rupture. Therefore, the ruptured vessels appeared as purpura on the cheeks.

### Case reports that showed an association between the Valsalva maneuver and the appearance of purpura on the skin

Limited case reports have shown a relationship between the Valsalva maneuver and the appearance of purpura. This case report discusses the purpura that developed on the cheeks of a 7-year-old following the performance of the Valsalva maneuver underwater. Two other case reports discussed the appearance of purpura resulting from forcefully breathing into balloons, where the forced expiration against a closed glottis led to the appearance of several rashes [3, 4]. Aside from holding the breath underwater and blowing forcefully into balloons, several different activities and stressors have been associated with the appearance of Valsalva purpura on the skin. Heavy powerlifting can be one activity that may lead to the development of Valsalva purpura. One case report talks about the appearance of a neck rash in a powerlifter who was doing several squats while lifting very heavy weights. The patient mentioned that the rash suddenly appeared during his weight training session and that he did not have any vascular disorders. He was reassured that such manifestations could result from increased intravascular capillary pressure and did not require further treatment. The same case report also talks about three different case reports that found an association between heavy weightlifting and the development of subarachnoid hemorrhage, which can also develop due to an increase in intravascular capillary pressure [11]. Another case report discussed the case of a 7-year-old boy who developed a rash behind his ears, which extended to his face and forehead. This boy had been playing with his friends and competing to see who could make their faces turn red. In the clinic, the boy demonstrated what he did in the competition, and it was a forced Valsalva maneuver, which explained the appearance of the rash [12]. A different case report also discussed a 7-year-old child who presented with a sudden, asymptomatic rash around her eyes. No trauma was noted, and all her examinations were unremarkable. However, the doctor noted that she received treatment for an upper respiratory tract infection associated with a non-productive cough. Therefore, they concluded that excessive coughing in this patient caused an increase in intravascular pressure [13]. All these case reports support the relationship between the Valsalva maneuver and the appearance of purpura, as the Valsalva maneuver can cause marked changes in the blood pressure and can raise the intra-abdominal and intra-thoracic pressure, leading to rupture of capillaries and manifesting as purpura.

### Bruising in children

Bruising happens due to the movement of blood from damaged blood vessels into subcutaneous tissues. Bruises are very common in children due to their playful nature. However, bruises can sometimes be more serious and may indicate a serious underlying hemostatic abnormality, such as an inherited bleeding disorder, or be a sign of non-accidental injury (NAI) [14]. The main medical causes of abrupt purpura in children include inherited or acquired coagulopathy. The most common acquired disorder of coagulation is immune thrombocytopenic purpura (ITP), whereas the most common inherited coagulation disorder is von Willebrand disease, which has an incidence of up to 1%, followed by factor VIII deficiency (hemophilia A) and factor IX deficiency (hemophilia B), which occur in 0.02% and 0.005% of live male births, respectively. Other medical conditions include infections (e.g., meningococcemia), malignancy (e.g., leukemia, neuroblastoma), nutritional deficiencies (e.g., vitamin K, vitamin C), severe systemic illness (e.g., disseminated intravascular coagulation), connective tissue disorders (e.g., Ehlers-Danlos syndrome, osteogenesis imperfecta) and autoimmune or inflammatory disorders (e.g., ITP, Henoch-Schoenlein purpura, Gardner-Diamond syndrome) [15]. Careful history taking, good physical examination, and relevant investigations are important to approach an accurate diagnosis. Some initial laboratory tests that must be considered include complete blood count, peripheral blood smear, prothrombin time, and partial thromboplastin time [16, 17].

## CONCLUSION

This case report highlights one of the unique presentations of purpura occurring on the face following the Valsalva maneuver in young children. By explaining the child's history and discussing different cases and causes of bruising, we have gained valuable insights into the potential risks and complications that can arise from breath-holding activities. This report shows the importance of recognizing different types of bruising as well as the potential consequences of seemingly harmless activities such as breath-holding or blowing balloons, particularly in children.

## Personal thanks

We would like to express our deepest appreciation to Dr. Alia Ali Galadari, whose dedication and hard work were instrumental in developing this case report. Her thorough research, insightful analysis, and meticulous attention to detail greatly enriched the content and quality of this paper. Additionally, we would like to sincerely thank Dr. Moteb Al Otaibi, Associate Professor of Dermatology at Unaizah University, College of Medicine, Qassim University, KSA, for sharing his expertise and providing the foundational case information. Dr Moteb’s insightful guidance and supervision were invaluable in shaping the direction and depth of this report.
